# Zebularine reactivates silenced *E-cadherin *but unlike 5-Azacytidine does not induce switching from latent to lytic Epstein-Barr virus infection in Burkitt's lymphoma Akata cells

**DOI:** 10.1186/1476-4598-6-3

**Published:** 2007-01-10

**Authors:** Sieta P Rao, Markus P Rechsteiner, Christoph Berger, Jürg A Sigrist, David Nadal, Michele Bernasconi

**Affiliations:** 1 Experimental Infectious Diseases and Cancer Research, University Children's Hospital, University of Zurich, August Forel Str. 1, CH-8008 Zürich, Switzerland

## Abstract

Epigenetic silencing of regulatory genes by aberrant methylation contributes to tumorigenesis. DNA methyltransferase inhibitors (DNMTI) represent promising new drugs for anti-cancer therapies. The DNMTI 5-Azacytidine is effective against myelodysplastic syndrome, but induces switching of latent to lytic Epstein-Barr virus (EBV) *in vitro *and results in EBV DNA demethylation with the potential of induction of lytic EBV *in vivo*. This is of considerable concern given that recurrent lytic EBV has been linked with an increased incidence of EBV-associated lymphomas. Based on the distinct properties of action we hypothesized that the newer DNMTI Zebularine might differ from 5-Azacytidine in its potential to induce switching from latent to lytic EBV. Here we show that both 5-Azacytidine and Zebularine are able to induce expression of *E-cadherin*, a cellular gene frequently silenced by hypermethylation in cancers, and thus demonstrate that both DNMTI are active in our experimental setting consisting of EBV-harboring Burkitt's lymphoma Akata cells. Quantification of mRNA expression of EBV genes revealed that 5-Azacytidine induces switching from latent to lytic EBV and, in addition, that the immediate-early lytic infection progresses to early and late lytic infection. Furthermore, 5-Azacytidine induced upregulation of the latent EBV genes *LMP2A, LMP2B, and EBNA2 *in a similar fashion as observed following switching of latent to lytic EBV upon cross-linking of the B-cell receptor. In striking contrast, Zebularine did not exhibit any effect neither on lytic nor on latent EBV gene expression. Thus, Zebularine might be safer than 5-Azacytidine for the treatment of cancers in EBV carriers and could also be applied against EBV-harboring tumors, since it does not induce switching from latent to lytic EBV which may result in secondary EBV-associated malignancies.

## Findings

Abnormal hypermethylation of the promoters of cancer-related or tumor suppressor genes is commonly found in primary neoplasms and tumor cell lines [1]. Thus, pharmacologic inhibition of DNA methylation could provide an effective means of epigenetic anti-cancer treatment. Indeed, 5-Azacytidine, a pyrimidine ring analogue of cytidine and DNA methylase inhibitor (DNMTI), has proven to be effective against myelodysplastic syndrome in a phase III randomized clinical trial [2]. 5-Azacytidine forms covalent complexes with cytosine- [C5]-specific DNA methyltransferases and inhibits their activity [3]. In addition, 5-Azacytidine is activated by uridine-cytidine kinase and can be incorporated into both RNA and DNA. Incorporation into RNA interferes with protein translation [4], which is the cause of 5-Azacytidine toxicity. This substance is also characterized by a low stability in aqueous solution [5,6].

Different types of cancers including Burkitt's lymphoma (BL) and nasopharyngeal carcinoma (NPC) harbor latent Epstein Barr virus (EBV) [7] and maintenance of latent EBV is partially mediated by hypermethylation of the EBV genome. Thus, it is not surprising that 5-Azacytidine induces switching of latent to lytic EBV *in vitro *[8-11] and results in EBV DNA demethylation in NPC patients with the potential of induction of lytic EBV [12]. Recurrent lytic EBV caused by chronic disruption of EBV latency due to long-lasting methotrexate treatment in EBV-carrying rheumatoid arthritis and polymyositis patients has been linked with an increased incidence of EBV-associated lymphomas [13]. Therefore, since DNMTI need to be administered for long periods of time to treat cancers, DNMTI with the potential to induce lytic EBV could have detrimental consequences in EBV carriers and be inappropriate to combat EBV-carrying tumors.

Zebularine (1-(β-D-ribofuranosyl)-1,2-dihydropyrimidin-2-one), a newer cytidine analog containing a 2-(1H)-pyrimidinone ring, acts as 5-Azacytidine by forming covalent complexes with DNMT [14], and in addition acts as transition state analog inhibitor of cytidine deaminase by binding covalently at the active site [15]. In comparison to 5-Azacytidine, Zebularine has little toxicity; shows increased stability [16,17], and targets preferentially tumor cells [18]. Hence, Zebularine promises to be a better drug than 5-Azacytidine for epigenetic therapy of cancer. Nevertheless, the potential of Zebularine in inducing lytic EBV is unknown.

Based on the distinct properties we hypothesized that Zebularine might differ from 5-Azacytidine in its potential to induce lytic EBV. Thus, we compared the effects of both DNMTI on EBV latency in the BL cell line Akata, a well-established model to study switching of latent to lytic EBV which also allows the study of DNMTI effects on cellular genes silenced in cancer cells.

**Figure 1:**
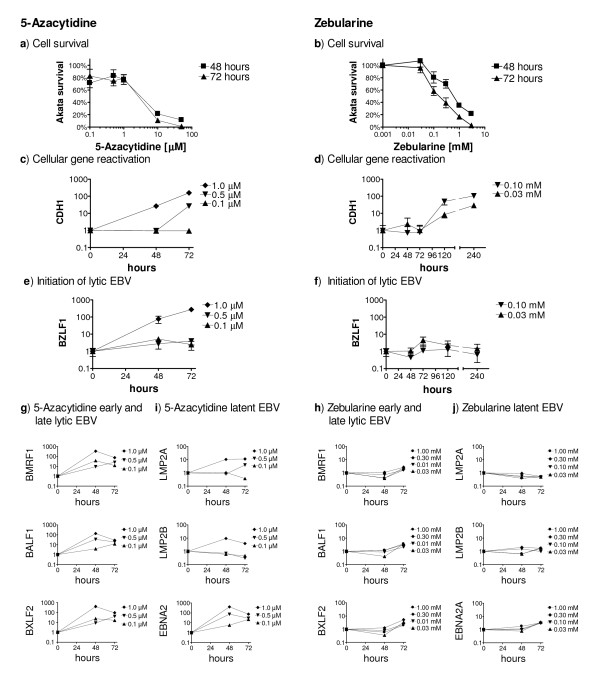
**Response of Burkitt's lymphoma Akata cells to 5-Azacytidine and Zebularine treatment. (a, b) Determination of non-toxic concentrations for Akata treatment ***in vitro *with 5-Azacytidine (a) and Zebularine (b). 5 × 10^5 ^viable cells were seeded in 1 ml medium with increasing 5-Azacytidine or Zebularine concentrations. Living cells (trypan-blue exclusion) were then counted after 48 h and 72 h. The highest non-toxic-concentrations are: 1 μM 5-Azacytidine (a) and 100 μM for Zebularine (b). **(c, d) Expression of *E-cadherin (CDH1) ***normalized to *HMBS*. Treatment with 1.0 μM 5-Azacytidine resulted in a 30-fold induction of *CDH1 *expression after 48 h (c), and with slower kinetics 100 μM Zebularine induced a 60-fold activation after 120 h and was sustained after 8 days of continuous Zebularine treatment (d). **(e, f) *BZLF1 *expression **indicating initiation of lytic EBV was observed after 1 μM 5-Azacytidine treatment (e), while 30 μM or 100 μM Zebularine do not activate *BZLF1 *expression, even after 8 days (f). Data in a-f are given as means ± SD from three independent experiments **(g, h) Expression of early and late lytic EBV genes**. Treatment with 5-Azacytidine results in increased expression of both EBV early and late antigens (g). Treatment with Zebularine did not show any significant increase of expression of early or late lytic antigens (h). **(i, j) Expression of latent EBV genes ***LMP2A *and *EBNA2 *increased around 10-fold upon treatment with 1.0 μM 5-Azacytidine. *EBNA2 *expression increased about 60-fold by 0.5 μM and 1.0 μM 5-Azacytidine at 48 h. The lowest 5-Azacytidine concentration used (0.1 μM) was also able to induce a 50-fold increase of *EBNA2 *expression at 72 h (i). *LMP2B *did show a maximal increase when treated with 1 μM 5-Azacytidine. (i). Treatment with Zebularine concentrations between 0.03 mM and 1 mM did not have a significant effect on *LMP2B *and *EBNA2 *gene expression up to 72 h (j). Data in g-j are given as means of three independent experiments.

We first determined the concentrations of 5-Azacytidine and Zebularine without cytotoxicity within 48 h. The highest sub-toxic concentration of 5-Azacytidine was 1 μM (Fig. 1a) and of Zebularine was between 0.03 mM and 0.1 mM (Fig. 1b).

To validate our experimental conditions we next measured in Akata cells the expression of cellular genes known to be reactivated by Zebularine in other tumor cell lines including *CDKN1A*, *CDKN1B *and *CDKN2A *genes [18,19]. Using quantitative real-time PCR (qRT-PCR), expression of these cyclin-dependent kinases (CDK) was already detectable in the non-treated cells and did not further increase upon treatment neither with 5-Azacytidine nor Zebularine (not shown) suggesting that in the p53 mutated Akata cells [20] these CDKs are not silenced by hypermethylation. This did not hold true for *E-cadherin *(*CDH1*). E-cadherin mediates cell adhesion and loss of its expression by hypermethylation [21] is responsible for increased cancer invasiveness and metastasis [22]. *CDH1 *expression is reactivated by 5-aza-2'-deoxycytidine treatment in the BL cell line Raji [23]. We therefore measured the expression of *CDH1 *by qRT-PCR in treated Akata cells (Fig. 1c and 1d). Expression of *CDH1 *was not detectable in non-treated Akata cells. Treatment with 1.0 μM 5-Azacytidine resulted in a 30-fold induction of *CDH1 *mRNA expression after 48 h, that increased to 150-fold at 72 h. At a concentration of 0.5 μM, 5-Azacytidine showed a maximal 30-fold activation of *CDH1 *after 72 h and at 0.1 μM had no significant effect on expression of *CDH1 *mRNA even after 72 h. Zebularine (100 μM) induced a 50-fold activation of *CDH1 *after 120 h (5 days), that was sustained and increased to 100-fold after 10 days of continuous Zebularine treatment. This delayed effect was expected since Zebularine follows a slower kinetic with maximal effects later than 5-Azacytidine also in other cellular backgrounds [18,24]. These results show that 5-Azacytidine and Zebularine are able to induce expression of *CDH1 *known to be frequently silenced by hypermethylation [22,25], and demonstrate that both DNMTI are active in our experimental setting.

The first step in activation of lytic EBV infection is the expression of the immediate-early lytic EBV gene *BZLF1*. Zta, the product of *BZLF1*, is a transcription factor that regulates the expression of early lytic EBV genes, and activates the lytic EBV gene expression program [26]. To determine the effects of 5-Azacytidine and Zebularine on switching latent to lytic EBV in Akata cells, we measured the mRNA expression of *BZLF1 *by qRT-PCR in Akata cells treated with increasing concentrations of 5-Azacytidine at 48 h and 72 h and for Zebularine at up to 10 days. 1 μM 5-Azacytidine was able to induce activation of *BZLF1 *mRNA expression (Fig 1e). Induction of *BZLF1 *mRNA was robust, reaching about 280-fold over mock-treated cells. 5-Azacytidine concentrations lower than 1 μM did not have a significant effect on *BZLF1 *mRNA expression. Cytotoxic concentrations higher than 1 μM also activated *BZLF1 *mRNA expression to even a greater extent (not shown). Treatment of Akata cells with 100 μM Zebularine did not result in induction of *BZLF1 *mRNA (Fig. 1f). Higher concentrations of Zebularine in the range of 1 mM to 3 mM resulted in a 10-fold induction of *BZLF1 *mRNA (not shown), likely due to stress response to cytotoxic insult, rather than specific activation of *BZLF1 *mRNA expression. These results confirm that 5-Azacytidine initiates lytic EBV infection at sub-toxic concentrations and in addition demonstrate that by contrast treatment with Zebularine up to 10 days does not provoke any change in *BZLF1 *mRNA expression levels in Akata cells.

To elucidate whether activation of the master regulatory lytic EBV gene *BZLF1 *by 5-Azacytidine is followed by activation of early and late lytic EBV genes and whether Zebularine does activate other lytic EBV genes independently of *BZLF1 *we also quantified mRNA expression of *BALF1*, *BMRF1*, and *BXLF2 *(Fig. 1g and 1h). *BALF1 *codes for an anti-apoptotic cytoplasmic early lytic antigen, *BMRF1 *for the early antigen-diffuse EA-D, and *BXLF2 *for the late lytic antigen glycoprotein gp85. Treatment of Akata cells with 1 μM 5-Azacytidine resulted in increased expression of both early and the late lytic antigens at 48 h with surprisingly similar kinetics. Even 0.1 μM 5-Azacytidine was able to increase mRNA expression of lytic EBV genes by a ten-fold at 72 h. By contrast, no expression of early or late lytic EBV genes was measurable in Akata cells treated with Zebularine. Even treatment with 1 mM Zebularine; a concentration that apparently did induce expression of *BZLF1 *mRNA after 48 h, was not able to activate mRNA expression of other lytic EBV genes. This supports our above mentioned interpretation that expression of *BZLF1 *mRNA at a 1 mM Zebularine dose was not a specific event with subsequent full lytic EBV gene expression. These results indicate that 5-Azacytidine activates all three phases of lytic EBV infection and evidence that Zebularine does not induce switching from latent to lytic EBV.

The failure of Zebularine to induce switching from latent to lytic EBV could be due to either having no effect on EBV at all or due to reinforcement of latent EBV gene expression. Thus, we compared the quantitative mRNA expression of latent EBV genes before and during treatment with 5-Azacytidine or Zebularine. Treatment of Akata cells with 1.0 μM and 0.5 μM 5-Azacytidine increased *LMP2A *mRNA expression by 10-fold at 48 h and at 72 h, respectively, and *LMP2B *mRNA by around 10-fold at 48 h (1.0 μM). The effects on *EBNA2 *mRNA expression were more marked with an increase by 60-fold using 5-Azacytidine at 0.5 μM and by 400-fold at 1.0 μM at 48 h. Even the lowest 5-Azacytidine concentration used (0.1 μM) was able to induce a 30-fold increase of *EBNA2 *mRNA expression at 72 h (Fig. 1i). We and others have previously shown a similar activation of *LMP2 *and *EBNA2 *upon induction of lytic EBV by IgG cross-linking in Akata cells [27,28]. In striking contrast, none of the tested Zebularine concentrations exhibited an effect on latent EBV gene expression (Fig. 1j), suggesting that the failure of Zebularine to induce switching of latent to lytic EBV is not due to reinforcement of latent EBV gene expression. We therefore conclude that Zebularine has no effect on EBV gene expression in Akata cells, neither on lytic nor latent.

In the BL cell line Akata, both 5-Azacytidine and Zebularine are able to reactivate an important regulatory gene like *E-cadherin*, which when silenced by hypermethylation contributes to cell malignancy. Hypermethylation is also an important regulatory mechanism of EBV latency. This is emphasized by the fact that EBV can modulate the activity of DNA methyltransferases through the latent membrane protein LMP1, that can induce activation of DNA methyltransferases in epithelial carcinoma and lead to silencing of *E-cadherin *expression [29]. 5-Azacytidine and Zebularine act by different mechanisms [24]. Importantly, here we show that Zebularine, unlike 5-Azacytidine, does not disrupt EBV latency. Thus, Zebularine might be safer than 5-Azacytidine for the epigenetic treatment of cancers in EBV carriers and could also be employed to treat EBV-harboring tumors, since it does not induce switching from latent to lytic EBV a process linked to secondary EBV-associated malignancies.

## Materials and methods

### Cell lines

The human BL cell line Akata was cultured in RPMI 1640 medium (Invitrogen, Basel, Switzerland), supplemented with 10% heat-inactivated fetal calf serum, L-Glutamine (1%), penicillin (1 U/ml), streptomycin (1 μg/ml). The human bladder carcinoma cell line T24 was cultured in McCoy's medium supplemented with 10% fetal calf serum, L-Glutamine (1%), penicillin (1 U/ml), streptomycin (1 μg/ml). The cells were grown at 37°C in 5% CO_2 _humidified atmosphere and split every third day. Akata cell line was a kind gift from Dr. A. Bell (Birmingham, UK), and T24 cell line was a kind gift from Dr. H. Wunderli-Allenspach (ETH Zürich, Switzerland). The optimal cell density (5 × 10^5 ^cells/ml) for experiments with Akata cells allowed logarithmic cell growth over the observation period.

### DNA-methylase inhibitor treatments

5-Azacytidine was purchased from Sigma-Aldrich Chemie Gmbh (Buchs, Switzerland) as lyophilized powder and stored at -20°C. 5-Azacytidine solution was prepared fresh for each experiment in PBS at a concentration of 2.4 mg/ml (10 mM) and sterile filtered. Zebularine was purchased from Calbiochem (Merk Biosciences, Darmstadt, Germany) and stored at 4°C. Zebularine was dissolved in PBS at a concentration 28.5 mg/ml (120 mM), sterile filtered and stored at 4°C. For each experiment with Akata cells, viable cells were counted by trypan-blue exclusion method and resuspended in fresh medium at 0.5 × 10^6 ^cells/ml. T24 cell line experiments were performed as described [18].

### Quantitative real-time PCR analysis

RNA isolation was then performed by using Rneasy Mini Kit (Qiagen, Hombrechtikon, Switzerland) according to the manufacturer's protocol. Contaminating genomic DNA was removed by using DNA-free (Ambion Europe, Huntingdon, UK) according to the manufacturer's protocol. The RNA concentration was measured with an Eppendorf Bio Photometer (Vaudaux Eppendorf, Dübendorf, Switzerland). cDNA was prepared by reverse transcription of total RNA using the Omniscript RT-Kit (Quiagen) following the manufacturer's protocol. qRT-PCR was performed in a reaction volume of 10 μl with the ABI-TaqMan Master Mix with uracil-N-glycosylase (Applied Biosystems, Rotkreuz, Switzerland). Sequence information for the Taqman systems will be furnished upon request. mRNA expression of the target genes was normalized to the expression of the housekeeping gene *HMBS *[30]. The normalized transcription values correspond to 2^-C^_T_^(EBV)-C^_T_^(HMBS) ^= 2^-ΔC^_T_, where C_T _is the cycle threshold number that quantifies the target present.

## Abbreviations

BL: Burkitt's lymphoma; EBV: Epstein-Barr virus; EBNA: EBV nuclear antigen; LMP: latent membrane protein; NPC: nasopharingeal carcinoma; qRT-PCR: quantitative real-time PCR; RT: retrotranscription.

## Competing interests

The author(s) declare that they have no competing interests.

## Authors' contributions

SPR carried out cell experiments and qRT-experiments and helped to draft the manuscript. MPR and MB carried out part of the cell experiments and qRT-experiments, participated in the experimental design and data analysis. CB designed and validated the qRT-PCR system for EBV. JAS carried out part of the cell experiments. DN and MB conceived the study, participated in its design and coordination, and drafted the manuscript. All authors read and approved the final manuscript.
